# The negative impact of chronic tobacco smoking on adult neuropsychological function: a cross-sectional study

**DOI:** 10.1186/s12889-021-11287-6

**Published:** 2021-06-30

**Authors:** Mohammed Sh. Nadar, Abdullah M. Hasan, Mohammed Alsaleh

**Affiliations:** 1 Occupational Therapy Department, Faculty of Allied Health Sciences, Kuwait University, Jabriah, Kuwait; 2 School of Pharmacy and Pharmaceutical Sciences, Cardiff University, Cardiff, UK

**Keywords:** Working memory, Cognitive impairment, Nicotine, Chronic smoking

## Abstract

**Background:**

The evidence on the effects of chronic tobacco smoking on neuropsychological functions is conflicting. The literature remains limited by inconsistent accounting for potentially confounding biomedical and psychiatric conditions. This study aimed to assess the neuropsychological functions of adult chronic tobacco smokers in comparison to group-matched non-smokers.

**Method:**

The study included 73 smokers and 84 group-matched non-smokers. The data was collected during the year 2019. After an initial interview to collect demographics and smoking profile, the subjects undertook neuropsychological assessments that targeted a wide range of cognitive domains.

**Results:**

The performance of smokers was poorer on almost all neuropsychological domains, namely selective attention (*p* ≤ .001, *p* = .044), alternating attention (*p* = .002) working memory (*p* ≤ .001), Short-term memory (*p* = .006 and .003), Long-term memory (*p* ≤ .001), processing accuracy (*p* ≤ .001), and executive function (*p* = .011 and .026). Smokers were intact on processing speed. Smoking accumulation and lower age onset of regular smoking were correlated with lower neuropsychological function.

**Conclusion:**

Our findings add to the growing body of evidence suggesting that chronic tobacco smoking impacts cognition negatively.

## Background

In 2015, around a quarter (24.9%) of the global population were current users of some form of tobacco [1]. Smoking is one form of tobacco exposure that is prevalent across the world.

The harmful impact of chronic tobacco smoking on physical health is well documented and includes cardiovascular diseases, respiratory diseases, and various forms of cancer [2]. Additionally, chronic smoking is implicated in the pathogenesis of neuropsychological dysfunction and has been directly linked to increased risk of depression and cognitive impairment [1, 3, 4].

A large number of studies have examined the effects of smoking on neuropsychological function across multiple variables. Compared to non-smokers, chronic smoking was cumulatively reported to have detrimental effects on various neuropsychological domains, including general intellectual abilities, processing speed, attention, memory, cognitive flexibility and executive functions [2, 3, 5]. The effects of smoking on cognition are believed to vary based on the dose and onset of regular use. Although the majority of studies provide evidence on the effect of chronic tobacco smoking on neuropsychological impairments, conflicting evidence also exists. For example, a 10-year longitudinal study of 1436 older adults found that smokers were less likely to develop cognitive impairment than those who had never smoked [6]. Another study consisting of 2553 adults found smokers to have lower rates of cognitive impairment compared to non-smokers [7], and both studies concluded that smoking may be protective of cognitive function. Several other studies also support the notion that cigarette smoking seems not to affect cognition or to have a positive effect on some aspects of cognitive function of smokers [8–11].

Based on the current literature, it is difficult to draw solid conclusions on the impact of smoking on neuropsychological function [2]. The influence of tobacco smoking on specific domains of cognition is complex and much remains to be known about its impact on neuropsychology and cognition. The literature remains limited by inconsistent accounting for potentially confounding biomedical and psychiatric conditions. For example, many of the studies did not account for confounding variables such as psychiatric disorders and comorbid substance abuse (i.e., alcohol, cannabis, and other drugs). In other studies, the duration of tobacco use was not taken into account [12]. Other studies varied in terms of which subcategories of specific neuropsychological domains were tested. Some controversy also remains regarding smoking effect on specific cognitive functions, and individual differences in smoking cognitive effects. Consequently, it is essential to continue to investigate the association between chronic tobacco smoking and potential neuropsychological impairments while controlling for possible confounding variables. This cross-sectional study *aimed* to assess the neuropsychological functions of chronic tobacco smokers in comparison to group-matched non-smokers. It was hypothesized that chronic tobacco smokers would have significantly poorer global neuropsychological functions compared to non-smokers.

## Method

### Participants and procedure

We recruited the participants via social medial and local adverts during the year 2019. The participants were interviewed regarding cigarette smoking and use of other tobacco products. We excluded participants who reported current use of other substances that are known to affect cognition (i.e., alcohol, cannabis, and psychotropic medications, except for caffeine) up to 3 months prior to study enrollment. Since psychiatric illness is strongly correlated to cognitive impairment, we excluded subjects with any known psychiatric problems or mental diseases. Participants with any medical condition or history of serious head injury that are known to influence cognition were also excluded. To be included in the smoking group, the participant must have been a smoker for at least 10 years and smoked a minimum of one pack per day. The smokers and non-smokers were matched as a group for age, sex, ethnicity, educational, and socioeconomic status. Past smokers and second-hand smokers were excluded from the non-smokers’ group.

The study was approved by the Institutional Review Board, and all participants signed a written consent form to participate in the study before data collection. An initial interview collected information related to socio-demographic, health, and smoking profiles. After the interview session, all participants completed a comprehensive battery of outcome measures assessing neuropsychological functions that target a wide range of cognitive domains. All the measures used in this study have well-established and comprehensive psychometric properties, and were used in other studies on neuropsychological function (a brief description of each measure is provided in Table 1). In addition to the neuropsychological measures, we used the Grooved Pegboard (Lafayette Instrument, Lafayette, IN) as a measure of fine motor dexterity, which requires visual-spatial and motor coordination. The test battery was administered by a trained researcher, and the entire battery required about 60–70 min to complete. The participants were allowed short breaks between tests, and smokers were free to smoke during the breaks if desired. The sequence of outcome measures was administered consistently across all participants.

**Table 1:** Assessments used in the study

No.	Measure	Description	Variables measured	Time to administer	Scoring
1	Montreal Cognitive Assessment (MoCA) [13]	A sensitive screening tool for detecting mild cognitive dysfunction	Assess several cognitive domains, including attention, concentration, executive functions, memory, language, visuospatial skills, abstraction, calculation, and orientation.	10 min	Highest score = 30. The cutoff score of 26 indicates normal cognition.
2	The Stroop Color and Word Test [14]	This executive function measure primarily assesses the ability to inhibit interference from distracting stimuli (i.e., selective attention) in a fixed time (i.e., processing speed). In this test, the subject is instructed to read the names of colors from three different conditions as fast as possible. In the first condition, the subject reads a list of color names printed in black ink (W = Word). Next, the subject is presented with a card containing shapes in different colors of ink, and the subject names the color of the ink (C = Color). In the third condition, the subject is given a card with color names written in an incongruent ink color (when the words and colors do not match). The subject is required to name the color of the ink instead of reading the word (CW = Color-Word). That is, the subject is required to inhibit the interference arising from reading the word (i.e., an automated task) and naming the color of the ink (i.e., an effortful task).	Selective attention and processing speed.	5 min	The number of correct answers in each condition (W, C, CW) in 45 s.
3	Comprehensive Trail making test (CTMT) [15]	The CTMT test uses a set of five standardized visual search and sequencing tasks (trials) to evaluate brain functions. The CTMT requires the participant to utilize visual scanning and sequencing abilities to draw lines to connect a series of stimuli (numbers or letters) in a specified order as fast as possible. We used Trial 1 (processing speed) and Trial 5 (alternating attention).	Processing speed and alternating attention	5 min	The primary score is the response time required to complete each trial. The norms for each trail are presented in the form of Percentile ranks and T-scores, which have a mean of 50 and a standard deviation of 10.
4	Wisconsin Card Sorting Test-64 (WCST-64) [16]	The WCST-64 is a measure of abstract thinking in which the participant has to classify cards with various geometric shapes in different colors and numbers according to different criteria (e.g., color, shape, or number). The participants are expected to accurately sort every response card with one of four stimulus cards through feedback (right or wrong) given to them based on a classification rule. The rule changes every few cards without prior notice, and the participant has to figure out the new rule. It is typical for the participant to start making one or more mistakes when the rule changes.	Executive function	10–15 min	Error scores, with lower scores indicating better executive function. Perseveration errors (number of errors where the participant has used the same rule as the previous choice) Non-perseveration errors (all remaining incorrect responses other than the Perseveration errors)
5	The Contextual Memory Test [17]	The participant is presented with pictures of 20 related objects for 90 s, as the items to be remembered. The participant is then requested to recall the objects immediately and again after 15 min.	Short-term memory and long-term memory	7 min	number of objects correctly recalled
6	The Digit Span Task [18]	In this test, the subjects are read a series of digits of increasing length and asked to repeat the same sequence back to the examiner in order (forward span) or reverse order (backward span). Forward span captures attention efficiency and short-term memory capacity. Backward span is an executive task particularly dependent on working memory.	short-term memory and working memory	10 min	The maximum number of forward or backward digits recalled without error
7	The d2 Test of Attention [19]	The participant is presented with a paper that has 14 rows, each comprised of 47 characters, for a total of 658 items. The participant is instructed to scan each row and cross out any letter “d” that has two dashes, which can be above or below the letter “d” in any order while ignoring/suppressing irrelevant distracters. The distractors are orthographically similar stimuli (the letters “p” or “d” with 1–4 dashes over and under each letter). The participant is allowed 20 s per row before moving to the next one, with no pauses allowed between rows	processing speed, selective attention, sustained attention, and quality of visual scanning performance in response to the discrimination of similar visual stimuli.	8 min	Total number of items processed (TN): The aggregated number of processed items (both correct and incorrect) from each raw. This is a measure of processing speed. Omission errors (OE): The number of missed correct items. This is a measure of processing accuracy (carelessness). Commission errors (CE): The number of incorrect items crossed out. This is a measure of processing accuracy (impulsivity), with lower scores indicating better function. Total number of errors (TE): Total number of commission and omission errors (OE + CE). Total performance (TP): The difference between the total number of items processed and the total number of errors (TN-TE). This is a measure of the balance between processing speed and accuracy of attention mechanisms.
8	Grooved Pegboard [20]	The grooved pegboard device is a symmetrical board with 25 slotted holes in a 5 × 5 matrix. In this test, the participant is instructed to rotate and place grooved metal pegs into holes with matching slots, which are angled in different directions. The pegs must be inserted one at a time and as quickly as possible with their dominant hand without practice trials	Fine motor dexterity	5 min	The time required to complete 25 pegs.

After completion of all measures, the participants were presented with two questions which they were required to answer by “Yes” or “No”. 1) “Do you believe that smoking increases the risk of physical health problems, such as getting heart disease, lung disease, stroke and cancer?”, and 2) “Do you believe that smoking increases the risk of cognitive health problems, such as reduced memory, attention, and concentration?”.

### Statistical analysis

All statistical analysis was performed with Statistical Package for the Social Sciences (SPSS – Windows version 25; SPSS Inc., Chicago, IL). Comparison between groups of participants in terms of demographic characteristics and quantitative outcome measures were performed using t-tests for normally distributed data. Where homogeneity of variance was violated in a given model (Levine’s test), we used the Mann-Whitney U test for skewed data. Two-tailed statistics were used, and statistical significance was set at *P* < 0.05. Cohen’s D effect sizes were reported when significant effects of group on a cognitive variable were identified. Pearson correlations were used to assess the influences of the smoking variables of accumulation (number of years smoking), Cigarettes/day, and Pack-Years of smoking and decline in cognitive function.

## Results

The final sample included 73 smokers (M = 52.1 years, SD = 7.2) and 84 non-smokers (M = 51.7 years, SD = 8.1). Independent t-tests showed that both groups were comparable across participant socio-demographic and health characteristics. Table 2 shows the general characteristics of the study population.

**Table 2:** Comparison of demographics and general characteristics between the smoking and nonsmoking groups

Variable	Smokers (***n*** = 73)	Non-smokers (***n*** = 84)	***P*** < .05
Age (years) - (mean, SD)	52.1 (7.2)	51.7 (8.1)	.424
Sex (M/F) %	61/12	69/15	.071
Education (years) - (mean, SD)	13.9 (3.5)	14.1 (3.8)	.732
Household income - [n, %]			
Low	11 [15.1]	14 [16.7]	.533
Moderate	53 [72.6]	59 [70.2]	
High	9 [12.3]	11 [13.1]	
Body Mass Index - (mean, SD)	26.9 (3.6)	27.1 (4.8)	.714
Daily portions of fruits and vegetables - (mean, SD)	3.1 (1.2)	3.2 (1.4)	.312
Physically active - [n, %]			
Low	26 [35.6]	25 [29.8]	.092
Moderate	39 [53.4]	47 [55.9]	
High	8 [11.0]	12 [14.3]	
Hours of sleep/night - (mean, SD)	7.9 (2.5)	8.1 (2.7)	.671
Diabetes Mellitus - [n, %]	16 [21.9]	15 [17.8]	.794
Coronary Heart Disease - [n, %]	7 [9.5]	3 [3.6]	.061
Hypertension - [n, %]	10 [13.7]	7 [8.3]	.102
**Smoking profile**			
Age onset of regular smoking	17.7 (4.3)	–	–
Accumulation (total lifetime years of smoking)	33.7 (11.4)	–	–
Cigarettes/day	28.4 (5.5)	–	–
Pack-years^a^	46.8 (14.3)	–	–

^a^ Pack-years is calculated as the packs of cigarettes smoked per day, multiplied by the duration of tobacco use in years

Data for all neuropsychological tasks are summarized in Table 3. In the Montreal Cognitive Assessment (MoCA), both groups performed above the cutoff score of 26/30, indicating global cognition scores within the “normal” range. Nonetheless, the smokers’ group scored significantly lower than the non-smokers’ group (*p* = .042) with a moderate effect size of .49.

**Table 3:** Results of neuropsychological measures

Neuropsychological Measures	Subtest	Variables measured	Smokers (Mean ± SD)	Non-smokers (Mean ± SD)	*P* < .05.	Cohen’s D
Montreal Cognitive Assessment (MoCA)	–	Global cognition	26.1 (2.3)	27.3 (2.6)	0.002	.49
The Stroop Color and Word Test	Word condition (W)	Processing speed	86.02 (14.24)	87.11 (12.56)	.078	–
	Color condition (C)	Processing speed	52.46 (12.14)	53.72 (13.7)	.097	–
	Color-word condition (CW)	Selective attention	31.63 (5.45)	36.41 (9.32)	≤ .001	.62
Comprehensive Trail making test (CTMT)	Trial 1	Processing speed	41.78 (14.57)	43.09 (11.15)	.055	–
	Trial 5	Alternating attention	39.34 (11.84)	44.14 (8.45)	.002	.47
Wisconsin Card Sorting Test-64 (WCST-64)	Perseveration errors^a^	Executive function	10.3 (6.4)	8.2 (5.8)	.011	.34
	Non-perseveration errors^a^	Executive function	7.9 (5.8)	6.6 (5.3)	.026	.23
Contextual Memory Test	Immediate recall	Short-term memory	13.01 (2.45)	13.97 (2.59)	.006	.38
	Delayed recall	Long-term memory	11.13 (2.61)	12.89 (2.72)	≤ .001	.66
The Digit Span Task	Digits Forward	Short-term memory	5.41 (1.34)	6.12 (2.11)	.003	.40
	Digits Backward	Working memory	3.02 (1.24)	3.86 (.98)	≤ .001	.75
The d2 Test of Attention	TN-Number of all items processed	Processing speed	386.07 (47.48)	391.62 (52.36)	0.052	–
	OE-Omission errors^a^	Processing accuracy Carelessness	7.96 (5.07)	5.26 (4.74)	≤ .001	.55
	CE-Commission errors^a^	Processing accuracy Impulsivity	2.43 (2.41)	1.27 (1.85)	≤ .001	.54
	TE-Total errors^a^	Processing accuracy	10.39 (6.34)	6.53 (5.13)	≤ .001	.67
	TP-Total performance (TN-TE)	Selective attention	375.68 (46.53)	385.09 (51.16)	.044	.19
Grooved Pegboard	Dominant Hand^a^	Fine motor dexterity	73.12 (10.4)	69.46 (9.24)	.007	.37

^a^ Lower scores are indicative of better performance

The Word condition (W) and Color condition (C) component of the Stroop Color and Word Test revealed no significant differences between both groups, demonstrating comparable processing speed abilities. The Color-word condition (CW) yielded a significant difference between groups (*p* ≤ .001, d = .62) demonstrating better selective attention of non-smokers. The Comprehensive Trail making test (CTMT) Trial 1 subtest, which measures processing speed, revealed no significant differences between groups, but the Trial 5 subtest revealed a significant difference (*p* = .002, d = .47) representing healthier alternating attention function of non-smokers. For the Wisconsin Card Sorting Test-64 (WCST-64), a small but significant effect size was detected in favor of the non-smokers’ group, showing a slightly better executive function capacity for non-smokers as evident by fewer Perseveration errors (*p* < .011, d = .34) and Non-perseveration errors (*p* < .026, d = 0.23).

In the Contextual Memory Test, the non-smokers’ group performed significantly better with small effect size for short-term memory (*p* = .006, d = .38), and with moderate effect size for long-term memory (*p* ≤ .001, d = .66). The Digit Span Task revealed comparable short-term memory results to the Contextual Memory Test, where the non-smokers’ group performed better than the smoking group (*p* = .003, d = .40). For working memory, a significant and large effect size of .75 was detected in favor of the non-smokers’ group (*p* ≤ .001).

The total number of items processed in the d2 Test of Attention was similar in both groups, indicating parallel processing speed. However, when factoring in the errors in performance, the smoking group had significantly poorer processing accuracy than their non-smokers’ counterpart in both measures of error (OE-Omission errors: *p* ≤ .001, d = .55, CE-Commission errors: *p* ≤ .001, d = .54, and TE-Total errors: *p* ≤ .001, d = .67). The overall TP-Total performance of the d2 Test of Attention was significant with a small effect size (*p* = .044, d = .19) reflecting superior selective attention ability of the non-smokers’ group. As per the psychomotor domain, the non-smokers’ group outperformed the smoking group in the Grooved Pegboard test of fine motor dexterity (*p* = .007, d = .37).

A set of Pearson correlations were conducted to explore the relationship between smoking variables with scores of the neuropsychological measures. Higher smoking accumulation (total lifetime years of smoking) was significantly correlated with the lower neuropsychological performance of selective attention of the Stroop Color and Word Test (*r* = − 0.58; *p* = .009), alternating attention of the Comprehensive Trail making test (CTMT) (*r* = − 0.32; *p* = .027), and working memory of the Digit Span task (*r* = − 0.57; *p* ≤ .001). There was an association between lifetime years of smoking and poorer performance on the Grooved Pegboard psychomotor measure of fine motor dexterity (*r* = −.41; *p* = .047). The age onset of regular smoking was also correlated with the lower performance in the same measures above; namely selective attention (*r* = − 0.41; *p* = .037), alternating attention (*r* = − 0.29; *p* = .048), working memory (*r* = − 0.62; *p* ≤ .001), and also processing accuracy of the d2 Test of Attention (*r* = − 0.46; *p* = .039). There were no other significant correlations between the number of cigarettes smoked per day or Pack-Years and neuropsychological performance.

When asking the participants about the effects of smoking on health, 100% of non-smokers and 92% of smokers believe that smoking has negative effects on physical health, while 37% of non-smokers and 19% of smokers believed that smoking has negative effects on cognitive health.

## Discussion

The current study aimed to assess and compare the neuropsychological functions of chronic tobacco smokers in comparison to non-smokers. As hypothesized, the results indicated that chronic tobacco smokers had significantly poorer neuropsychological functions compared to their group-matched non-smokers. The poorer performance was apparent in almost all cognitive domains, namely attention, memory, processing accuracy, and executive function, but not processing speed.

With respect to global neuropsychological function, the performance of smokers in the Montreal Cognitive Assessment (MoCA) was not impaired since they scored within the “normal” range. Nonetheless, their performance was significantly weaker compared to their matched non-smokers. Despite the “normal” results of the MoCA, the more dedicated measures in this study clearly revealed a sub-optimal neuropsychological performance of our smokers’ group.

The effects of smoking on memory performance is inconsistent in the literature, with studies reporting significant differences between smokers and non-smokers in some memory measures [4, 21–25] while others reporting insignificant differences [10, 21, 22, 26–28]. Our results showed the neuropsychological domain of memory to be affected in our smoking sample and with group differences of moderate to strong effect sizes, as our non-smokers’ group outperformed the smokers in all components of memory measures. The non-smokers’ group had better short-term and long-term memory as indicated by their superior capacity to recall information in the Contextual Memory Test, which involves visual presentation of pictures, as well as the Digit Span Task, which involves auditory and verbal presentation of numbers. Working memory was particularly compromised in our chronic tobacco smokers, as evident by the largest effect size (d = .75) amongst the neuropsychological measures employed in this study. Similar results for working memory impairments were found in middle-aged adults [22, 29], young adults [30–33] and even adolescent smokers [34]. Based on the compromised overall memory performance of smokers, it is plausible to state that chronic tobacco smoking may predispose the development of dementia.

While the effects of tobacco smoking on memory have been widely studied, their effects on attention have been less investigated. Attention is central for learning and memory since encoding information requires attention in the first place. The smokers in our study were less able to block irrelevant information and focus their selective attention on the task at hand. This was strongly demonstrated in the Stroop Color and Word Test and less significantly demonstrated in the d2 Test of Attention. Alternating attention was also affected in our smokers’ CTMT test, indicating more difficulty to disengage and reengage the focus of attention in response to environmental stimuli, in comparison to the non-smokers’ group. Our findings on the domain of attention are consistent with the literature where attention was found to be affected in smokers [28, 31], but contrasted several other studies where attention was not affected [10, 26, 33, 34]. Given the intertwined relationship between attention and memory, it is reasonable to suggest that smoking may diminish memory as a result of decreasing attentional capacities, reflected in reduced ability to resist distraction and blocking irrelevant stimuli.

Executive function involves the simultaneous use of a set of cognitive abilities to allow the individual to perform higher-level complex tasks. The findings in our study detected a significant executive function difference in favor of the non-smokers’ group, who performed better in both subtests of the WCST-64. These results are consistent with previous literature findings of executive function limitation among smokers [9, 10, 22, 23, 25, 26, 31, 35], indicating that chronic tobacco smokers have inferior mental flexibility and abstract thinking compared to non-smokers.

Interestingly, processing speed was not significantly affected in the smoking group across all the four subtests we used in this study, albeit two of the subtests were statistically borderline (*p* = .052, .055). These findings agree with few studies [10, 26, 31, 33], but contrast with most other studies that assessed processing speed for their smoking participants [4, 21, 22, 36–38]. Despite this finding, the processing accuracy of our smokers’ sample was significantly lower than non-smokers (*p* ≤ .001). That is, the smokers’ processing speed matched that of non-smokers, but with a significantly higher number of errors. The substandard performance of smokers on measures of processing accuracy, as evident by a significantly higher number of cognitive errors, should be added to a growing list of neuropsychological sequelae associated with persistent smoking.

Within the smoker group, smoking onset was correlated with lower performance on working memory, selective attention, alternating attention, and the number of errors made. This means that individuals who start smoking at a younger age are at a greater risk of developing neuropsychological dysfunction. The impairments appear to manifest very early in smokers, as demonstrated in the inferior working memory of adolescents with a mean of only 4 years of smoking [34]. Additional support on the effects of smoking on the young brain was objectively shown in functional magnetic resonance imaging where young adult smokers had reduced prefrontal cortex activation during attentional tasks when compared with non-smokers [39]. This pronounced negative effects of smoking on the young brain might be explained by the fact that the prefrontal cortex has not completed its maturation, as the prefrontal cortex is one of the last brain areas to mature and is still developing during adolescence and early adulthood [40]. This stage of ongoing development makes the brain more susceptible to the influence of tobacco smoking and other psychoactive substances [40].

Correspondingly, smoking accumulation in this study similarly affected neuropsychological function, meaning the longer an individual smoked during their lifetime, the more prone they become to cognitive dysfunction. These results are consistent with other studies that reported total lifetime years of smoking to be correlated with inferior neuropsychological efficiency [22] and executive function [27]. Interestingly, the number of cigarettes per day or pack-years were not associated with neuropsychological performance in this study, contrasting the main findings of a recent review conducted by Conti et al. (2019), in which several studies included in their review reported a negative link between the number of pack-years and neuropsychological function [2]. This discrepancy in results may be confounded by the considerable variations in the number of pack-years reported across studies (ranging from 4.26 to 73.73) [2].

The magnitude of differences between smokers and non-smokers in this study extend beyond neuropsychological function to include the psychomotor domain as well. Our non-smokers’ group outperformed the smoking group in the Grooved Pegboard test of fine motor dexterity, albeit with a relatively small effect size of .37. Durazzo et al. [22] also reported significantly poorer fine motor dexterity performance in their smoking sample, but with much larger effect sizes (i.e., .72). The psychomotor function was also negatively correlated with smoking accumulation, where longer lifetime smoking was associated with reduced fine motor dexterity.

The question arises related to the reasons for heterogeneity in study outcomes between studies of comparable designs. For example, processing speed was reported in some studies to be reduced among smokers [4, 21, 22, 36–38] but not in other studies [10, 26, 31, 33]. The same point can be raised for memory and attention. Several reasons could explain such variations in the performance; 1) The outcome measures used to assess the target variable (i.e., processing speed) are different across studies, which may vary in their sensitivity and other psychometric properties. 2) Some studies did not report which version of the tests they used (e.g., original version, modified version, pen and paper, or electronic version). Depending on the assessment used, it could involve a visual task, an auditory task, or even a motor task. An assessment that involves the use of pen and paper will recruit a variety of motor and cognitive pathways in the brain to facilitate writing or tracing, while an electronic version of the same assessment will involve different brain pathways. Such variations in assessment components could yield different results. These factors make it less accurate to perform cross-study comparisons or to compare the study scores with normative data. A possible solution to help makes the results more comparable across studies, and thus more clinically meaningful, is to use complete reporting of the specific testing procedures (e.g., test version, subtests used, and scoring methods) and to use consistent and systematic data collection and analysis procedures.

The participants awareness about the negative effects of smoking on physical health were very high. However, their awareness about the negative effects of smoking on neuropsychological health was low, particularly among smokes (37% of non-smokers and 19% of smokers). This very low awareness of the negative consequences of smoking on neuropsychological function among smokers and non-smokers is a major public health issue that should be properly addressed.

Since tobacco smoking remains highly prevalent across the globe and is known to be influenced by public perception of risk and associations with negative outcomes [31], it is imperative to invest further in policy initiatives to control smoking. The emphasis of public health campaigns primarily focuses on physical health, and less commonly address neuropsychological health. Increasing public awareness should go beyond the already established physical health consequences to include the negative impact of smoking on neuropsychological function. We hope that raising awareness about the wider effects of tobacco smoking on cognition could help encourage people to stop smoking.

### Limitations and future research

There are several limitations and areas for improvement in future research. Although we attempted group matching for demographics and health variables, the self-reported health & medical profile can be problematic due to matters of inaccuracy of some participants. Future research should adopt standardized screening measures to provide accurate objective measures. The scope of neuropsychological impairment associated with chronic cigarette smoking has yet to be fully delineated. Large-scale prospective studies with more consistent, highly sensitive, and robust cognitive outcome measures are required to determine the true links between smoking and neuropsychological dysfunction.

## Conclusion

Chronic tobacco smoking seems to be a prospective risk factor for neuropsychological impairment, as expressed in our data by reduced attention, memory, executive function, and processing accuracy of smokers compared to their group-matched non-smokers. The cognitive performance of participants decreased as the number of years smoking increased. Similarly, the younger the age when regular smoking started, the lower was the cognitive performance. The consequences of smoking go beyond neuropsychological performance to encompass fine motor dexterity tasks as well.

## Data Availability

The datasets used during the current study are available from the corresponding author on reasonable request.

## Abbreviations

MoCA: Montreal Cognitive Assessment
CTMT: Comprehensive Trail making test
WCST-64: Wisconsin Card Sorting Test-64
